# Internet gambling disorder in adolescents: Prevalence and associated factors; A cross-sectional study of 360 cases

**DOI:** 10.1192/j.eurpsy.2023.1537

**Published:** 2023-07-19

**Authors:** M. Chaabane, D. Ben Touhemi, K. Chiha, W. Kammoun, J. Boudabous, I. Hajkacem, H. Ayadi, K. Khemakhem, Y. Moalla

**Affiliations:** Department of Child Psychiatry, Hedi Chaker Hospital, Sfax, Tunisia

## Abstract

**Introduction:**

Gambling disorders have increased over time due to the easy availability of online games.

**Objectives:**

The purpose of this study is to determine the prevalence of internet gambling disorder in an adolescent population and to identify associated factors.

**Methods:**

It was a cross-sectional, descriptive and analytical study, conducted among a sample of high school students, randomly collected in 6 schools in the region of Sfax during the month of February 2022. A pre-established form of 33 questions, including socio-demographic and family information was used.

The Arabic version of the Internet Gaming Disorder-20 (IGD-20) questionnaire was used to assess online gaming activity. It is a 20-item questionnaire on a five-point Likert scale ranging from 1 to 5 (strongly disagree to strongly agree). A respondent’s score was obtained by aggregating the 20 items. The higher the score, the more severe the gambling disorder. The cut-off score for the IGD-20 is 70. A score below 50 indicates occasional use; a score between 50 and 70 indicates problematic use; and a score above 70 indicates an online gambling disorder.

**Results:**

We collected 360 adolescents, 52.2% of whom were male. The mean age of our patients was 16.62 years.

A total of 4.7% of the adolescents had an online gambling disorder, 26.9% had problematic use, while 68.3% were occasional users.

The analytical study revealed that online video game addiction was associated with male gender (p ꞊0.003), the presence of relationship problems with parents (p ꞊0.000), and low academic achievement (p ꞊0.000).

**Conclusions:**

We draw attention to the necessary debate between sensible and problematic use of new technologies and the need for longitudinal prevention in schools.

**Disclosure of Interest:**

None Declared

